# Decomposing socioeconomic inequality in skilled birth attendant utilization in Ethiopia: A secondary data analysis

**DOI:** 10.1371/journal.pone.0327519

**Published:** 2025-09-17

**Authors:** Asebe Hagos, Melak Jejaw, Tesfahun Zemene Tafere, Misganaw Guadie Tiruneh, Getachew Teshale, Kaleb Assegid Demissie

**Affiliations:** Department of Health Systems and Policy, Institute of Public Health, College of Medicine and Health Science, University of Gondar, Gondar, Ethiopia; National Institute of Public Health: Instituto Nacional de Salud Publica, MEXICO

## Abstract

**Background:**

Inequalities in access to and utilization of skilled birth attendants (SBA) present a substantial challenge in low- and middle-income countries, hindering progress towards achieving universal health coverage in maternal health. Countries should regularly monitor inequality in access to SBA at both national and subnational levels. Therefore, this study aimed to measure socioeconomic inequalities and the contributing factors to the utilization of SBA among postpartum women in Ethiopia.

**Method:**

We used secondary data from the second cohort’s 6-week postpartum survey in combination with the baseline data of the Performance Monitoring for Action Ethiopia longitudinal study, conducted between November 2021 and October 2022. The study employed a multistage stratified cluster sampling technique to select 1,966 postpartum women. Socioeconomic inequality in the utilization of SBA was measured using the Erreygers Normalized Concentration Index (ECI) and visualized by a concentration curve. A concentration index decomposition analysis was conducted to identify the factors that contribute to the socioeconomic related health inequality in the utilization of SBA.

**Result:**

Utilization of SBA was 61.6% (95% CI: 59.0–64.0) in Ethiopia. The concentration curve in the utilization of SBA lay below the line of equality, and the ECI was 0.5308, with a standard error of 0.0398 and a p value < 0.001, demonstrating that the utilization of SBA was disproportionally concentrated among women belonging to the highest socioeconomic status. The concentration index decomposition analysis showed that household wealth index (40.3%), educational level (16.5%), place of residence (16.5%), antenatal care visits (15.7%), administrative regions (5.3%), and use of maternal waiting homes (2.9%) were the contributing factors to the socioeconomic inequality in the utilization of SBA in Ethiopia.

**Conclusion:**

We found a strong pro-rich socioeconomic related health inequality in the utilization of SBA in Ethiopia. Therefore, the government and responsible stakeholders need to implement targeted interventions such as improving health literacy, improving the coverage of antenatal care four visits, promoting the utilization of maternal waiting homes, and establishing financial support mechanisms for economically disadvantaged women to reduce the observed socioeconomic related health inequality in utilizing SBA.

## Introduction

In 2020, an estimated 287,000 women worldwide died from maternal causes, equal to nearly 800 deaths daily, or approximately one every two minutes [1]. Between 2000 and 2020, the global maternal mortality ratio (MMR) reduced from 339 to 223 per 100,000, representing a decrease of 34%. Nearly 95% of maternal deaths occurred in low and lower middle-income countries [1]. Sub-Saharan Africa alone accounted for about 70% (202,000) of these deaths, while Southern Asia contributed around 16% (47,000) [1,2]. In the same year, four countries were responsible for nearly half (49.7%) of the world’s maternal deaths: Nigeria (82,200), India (24,000), Democratic Republic of Congo (22,000), and Ethiopia (10,000). These countries represent 28.5%, 8.3%, 7.5%, and 3.6% of global maternal deaths, respectively [1].

The majority of maternal deaths are preventable with effective maternal health interventions [3]. A study conducted in 2018 reported that the leading causes of maternal mortality in Ethiopia were obstetric complications such as hemorrhage, obstructed labor, pregnancy induced hypertension, puerperal sepsis, and unsafe abortion [4]. Maternal health services provided by skilled healthcare professionals before, during, and after childbirth can greatly reduce maternal and newborn mortality rates [3]. The presence of skilled health care provider during childbirth plays an indispensable role in saving the lives of both mothers and newborns. Countries with a higher rate of maternal and neonatal mortality often have low coverage of SBA [5].

According to the United Nations Interagency Maternal Mortality Ratio estimation, the MMR in Ethiopia decreased from 953 deaths in 2000–267 deaths per 100,000 live births in 2020 [1]. The provision of skilled delivery services and improved access to emergency obstetrics care have played a crucial role in significantly reducing maternal deaths in the country [6].

The 2022/2023 National Health Equity Survey reported that the national coverage of SBA was 59%. Sidama region had the lowest coverage at 43%, while Addis Ababa city had the highest at 99%, indicating substantial regional disparities in the utilization of SBA [7]. In Ethiopia, the provision of skilled birth attendant services faces two main challenges. Firstly, although there has been progress, the coverage remains below both national and global targets [3,8]. Secondly, there is an unequal distribution of SBA services across different socioeconomic groups and geographic areas [7,9–13].

Health inequalities are apparent differences in health determinants and outcomes among subgroups within a population, based on demographic, geographic, and socioeconomic factors such as age, economic status, education level, sex, and place of residence [14]. The issue of inequality in health is a key national and global priority and a fundamental aspect of the Sustainable Development Goals (SDGs) [15]. Inequalities in access to and utilization of SBA present a substantial challenge in low- and middle-income countries, hindering progress towards achieving universal health coverage in maternal health [15,16].

In Ethiopia, previous studies have reported significant inequalities in the utilization of SBA, based on wealth status and education level [7,11,17,18]. Wealthier and more educated women had higher access to SBA compared to those from lower socioeconomic backgrounds [10,11,13,19]. There is also consistent evidence from various studies highlighting significant inequalities in the utilization of SBA across different regions of Ethiopia [7,10]. Additionally, there is a marked difference in access to and utilization of SBA between rural and urban women in the country [7,11,19].

To achieve universal health coverage in an equitable and progressive manner, countries should routinely evaluate the level of inequality in accessing SBA at both national and subnational levels [3,20]. Examining data beyond national averages uncovers disparities in health outcomes among different socioeconomic groups and geographic regions, as national averages can mask such variations. [20].

Monitoring the level of national health inequality provides valuable evidence to support the development of policies and strategies aimed at addressing health inequality [21]. However, the majority of existing studies are based on the 2016 Ethiopia demographic and health survey data, none of which reflect the present status of socioeconomic inequality in Ethiopia [10–13]. Even recent studies do not address the factors contributing to socioeconomic inequality in SBA [7]*.* Therefore, to address these gaps, this study aimed to measure socioeconomic inequalities using recent data and investigate the contributing factors to the utilization of SBA among postpartum women in Ethiopia. Findings from this study will assist policymakers and health managers in designing evidence-based policies and implementing effective interventions to reduce inequalities in the utilization of SBA services in Ethiopia.

## Methods

### Study settings, design, and data

Ethiopia, located in eastern Africa, is a landlocked nation with a total area of 1,104,300 km². It is the second most populous country on the continent, with an estimated population of 129.7 million. Of these, approximately 78%, live in rural areas [22]. During the data collection period, Ethiopia had ten administrative regions: Tigray, Afar, Amhara, Oromia, Somali, Benishangul-Gumuz, Southern Nations, Nationalities, and Peoples’ (SNNP), Gambella, Harari, and Sidama. Additionally, it has two city administrations: Addis Ababa and Dire Dawa.

Ethiopia’s healthcare system is organized into three tiers: primary (health posts, health centers, and primary hospitals), secondary (general hospitals), and tertiary (specialized hospitals) [23]. In the study regions, most communities have access to at least one health post, which serves as the most peripheral health unit. However, these health posts are staffed by health extension workers who primarily provide preventive and limited curative care, rather than skilled birth attendance [24]. Skilled birth attendance services are primarily available at health centers and all levels of hospitals, but not all women can access these facilities due to geographic and socioeconomic factors, or cultural barriers [25,26].

We employed data from the Performance Monitoring for Action (PMA) Ethiopia dataset. PMA is a survey project intended to produce data on various reproductive, maternal, and newborn health indicators [27]. The PMA data plays a great role in addressing the data gap in this priority area. PMA Ethiopia conducted a longitudinal survey that enrolled and followed pregnant women at six-week, six months, and one year postpartum. The longitudinal data was collected from two cohorts of women, from the periods 2019–2021 and 2021–2023 [28]. For the present analysis, we combined data from the six-week postpartum survey of the second cohort with the baseline data. This six-week dataset holds vital maternal health information that directly addresses our research question. The six-week survey was conducted from November 2021 to October 2022. To construct a complete dataset, we merged the six-week follow-up data with the baseline data from the second panel cohort using participant identification numbers [29]. The study employed a multistage stratified cluster sampling technique [27]. In the first stage, the major regions: Amhara, Oromia, SNNP and Addis Ababa city were selected, representing approximately 80% of the total population [30]. In the second stage, Amhara, Oromia, and SNNP were stratified into urban and rural strata, while Addis Ababa was considered as urban stratum. A total of 162 enumeration areas were then selected through simple random sampling, using a Central Statistical Agency’s sampling frame, with proportional allocation based on the size of each stratum. In the third stage, a fixed sample of 35 households was randomly selected from each enumeration area [27].

### Study population and sample size

**Fig 1:**
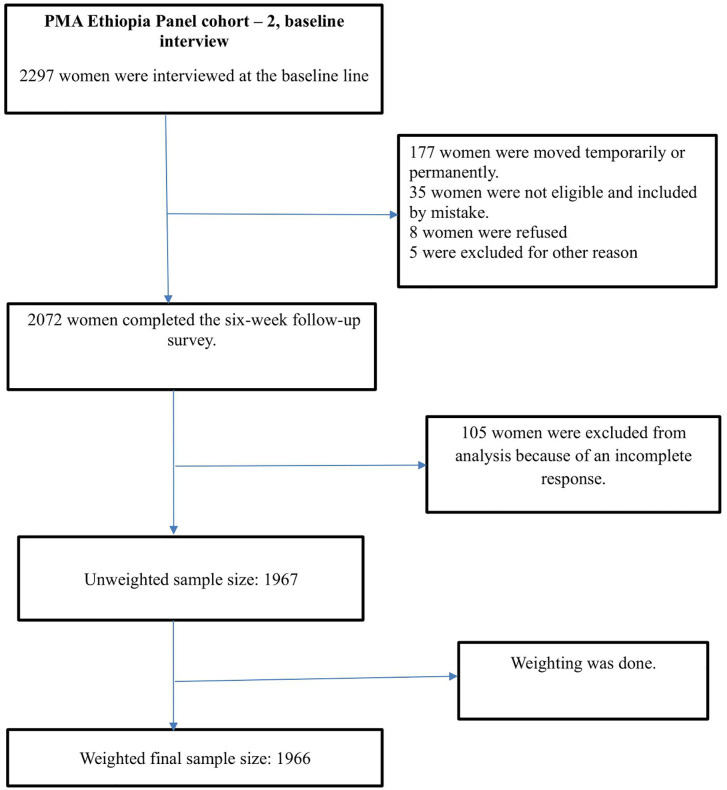
The final sample size and a schematic presentation of participants selection in the skilled birth attendant utilization in Ethiopia, PMA Ethiopia 2022.

Fig 1 presents the sample size of study that included a weighted sample of 1,966 women, with 92 from Addis Ababa, 413 from the Amhara region, 1,029 from the Oromia region, and 432 from the SNNP region. Women who were 5–9 weeks postpartum at the baseline survey, those who were 0–4 weeks postpartum and consented to both the baseline and follow-up surveys, and pregnant women at the start of the study who consented to participate in both the baseline and follow-up surveys were eligible for the 6-week follow-up survey [28,29].

### Data collection

Data were collected using the Open Data Kit (ODK) system on tablet computers. Before data collection, all forms undergo several quality checks to ensure the accuracy in representing the original paper questionnaire. PMA Ethiopia conducted a two-week training program for the supervisors and resident enumerators (data collectors). This training involved an in-depth review of survey protocols, questionnaire content, and interview skills. The training program included classroom exercises as well as three days of field practice. During the field practice, resident enumerators (data collectors) used ODK tools on their mobile phones to gain practical experience in data collection. The details are available in another source [27].

During the baseline survey, data collectors collected information on women’s sociodemographic characteristics, including age, education, region, parity, residence, marital status, household wealth status, and migrant status, as well as fertility preferences and birth histories. For women who were pregnant at the time of the survey, additional data was collected on estimated gestational age and utilization of maternal health services [27,28]. Likewise, during the six-week postpartum interview, data were collected on key maternal, neonatal, and delivery services. This included details on the receipt, timing, and specific components of antenatal care (ANC), delivery-related information, and the receipt of immediate postpartum services for both the mother and newborn.

### Study variables

The outcome variable of the study is the utilization of skilled birth attendant. Women are coded as 1 if assisted by doctors, nurses, midwives, and health officers and 0 otherwise.

Age of the mother (15–24, 25–34, 35–49), education level of the mother (no education, primary and secondary education, higher education), respondent’s religion (Orthodox, Protestant, Muslim, others), place of residence (urban, rural), antenatal care four or more visits (yes, no), marital status (single, married), administrative regions of Ethiopia (Addis Ababa city administration, Amhara, Oromia, SNNP), use of maternal waiting homes (MWHs) (yes, no), and household wealth index were explanatory variables of the study.

To calculate concentration indices, socioeconomic indicators such as income, wealth index, or education level are needed [31]. In this study, we used the wealth index as a measure of respondents’ socioeconomic status [31,32]. The wealth index of the household was developed using various indicators that are considered to be associated with a household’s wealth status. These indicators include ownership of household items (such as electric city, television, radio, watch, telephone, refrigerator), types of vehicles, water supply and sanitation (including the source of drinking water, type of toilet, and sharing of toilet facilities), housing conditions (such as material of floor, wall, roof), ownership of agricultural land, and types and the number of animals owned [33]. The wealth index of the household was constructed using principal component analysis. Subsequently, the household’s wealth index was categorized into five quintiles: quintile 1 (poorest), quintile 2, quintile 3, quintile 4, and quintile 5 (richest).

### Statistical analysis

#### Measuring socioeconomic inequality in skilled birth attendants.

Socioeconomic inequality in the utilization of SBA was assessed using the concentration curve (CC) and concentration index (CI). The concentration curve plots the cumulative proportion of SBA on the y-axis against the cumulative proportion of the population, ranked by wealth index from the poorest to the richest, in the x-axis. If SBA are evenly distributed among socioeconomic groups, the concentration curve will coincide with the line of equality. Conversely, if the SBA is concentrated in the higher socioeconomic groups, the concentration curve will lie below the line of equality [34,35]. The concentration curve assesses the existence of socioeconomic inequality in health care utilization among different socioeconomic strata but does not measure the magnitude of the inequality. The magnitude of inequality is measured by the concentration index. The concentration index, defined as twice the area between the concentration curves and the line of equality [34,36]. Hence, we used, concentration index to measure the degree of inequality in the use of SBA across socioeconomic strata.

The standard concentration index is best suited for health indicators measured on a ratio scale. However, since the outcome variable in this study is not measured on a ratio scale, the standard concentration index is not a proper measure of health-related inequality [37]. Therefore, we used the Erreygers normalized concentration index (ECI), which is most appropriate when dealing with binary health outcomes such as measuring inequality in the utilization of SBA [38].

As presented in equation 1, the ECI can be estimated as follows:


$$ECI\;=\;4^{*}\;\mu^{*}\;CI^{*}\;(y)\mathrm{\backslash ]}$$ (1)

Where ECI is the Erreygers normalized concentration index, μ is the mean of utilization of SBA, and CI is the generalized concentration index. The ECI ranges from −1 to +1. A positive ECI value indicates that the utilization of SBA is disproportionally concentrated among the wealthiest group (pro-rich), while a negative ECI value of CI indicates it is disproportionally concentrated among the poorest (pro-poor). A larger absolute of ECI suggests greater socioeconomic related health inequalities in the utilization of SBA. An ECI value of 0 indicates no socioeconomic inequality in the utilization of SBA [34].

#### Decomposition analysis of skilled birth attendant utilization.

After measuring the ECI, a decomposition analysis of the ECI was performed to identify the factors contributing to socioeconomic related health inequality in the utilization of SBA.

Any linear additive regression model concerning the health outcome variable y [34] is shown in equation 2.


$$\begin{array}{c} \text{y }=\;\alpha \;+\;\sum {\mathrm{\backslash nolimits}}_{\dot{k}}\beta_{k}x_{k}+\varepsilon \end{array}$$ (2)

In equation 2 “y” represents the health outcome variable (in this case, utilization of SBA), $x_{k}$ is a set of the socioeconomic determinants of the outcome variable, α is the intercept, and $\beta_{k}$ is the coefficient of $x_{k}\;$and, ε is the residual of the error term.

The concentration index for the outcome variable y, CI, is given in equation 3.


$$\begin{array}{c} CI\;=\;\sum {\mathrm{\backslash nolimits}}_{k}\left(\frac{\beta_{k}{\overset{―}{x}}_{k}}{\mu }\right)CI_{k}+\frac{GC_{\varepsilon }}{\mu } \end{array}$$ (3)

Where “CI” is the overall concentration index, μ denotes the mean of the health outcome variable, ${\overset{―}{x}}_{k}$ denotes the mean of $x_{k}$ (determinants), $CI_{k}$ is the concentration index for $x_{k}$, and $GC_{\varepsilon }$ represents the generalized concentration index for ε. Equation 3 consists of two components: the explained component and the unexplained component. The explained component, represented by $(\sum_{k}\left(\frac{\beta_{k}{\overset{―}{x}}_{k}}{\mu }\right)CI_{k}$), suggests the contribution of each explanatory variable to the socioeconomic inequality in the utilization of SBA, whereas, the unexplained (residual) part, denoted by ($\frac{GC_{\varepsilon }}{\mu }$), indicates the socioeconomic inequality in the utilization of SBA that cannot be explained by systematic variations across wealth index groups in the $x_{k}\;$. Ideally, this unexplained component should be near zero in a well specified model [34].

The decomposition analysis was originally designed for a linear, additively separable model, relying on the assumption of linearity. However, our study variable (utilization of SBA) is binary, so non-linear estimation is required. When handling a discrete change from 0 to 1, one option is to employ partial or marginal effects, which provide the change in estimated probability corresponding to a unit change in an explanatory variable [39]. Employing marginal effects offers an approximation of the non-linear relationship in the decomposition of concentration index [34,39]. Marginal or partial effects have been examined in analyzing inequalities within the health sector under non-linear conditions [39,40].

Equation 4 represents a linear approximation of the non-linear estimations.


$$\begin{array}{c} \text{CI }=\;\sum {\mathrm{\backslash nolimits}}_{k}\left(\frac{\beta_{\dot{k}}^{m},\;{\overset{―}{x}}_{k}}{\mu }\right)CI_{k}+\frac{GC\varepsilon }{\mu } \end{array}$$ (4)

Where $\beta_{\dot{k}}^{m}$, is the marginal effect of the explanatory variable $x_{k}$(dy/dxk).

Marginal effects can be derived from logit and probit regression models [41]. In this study we used the Generalized Linear Model (GLM) in the decomposition of concentration index in the utilization of SBA. The GLM approach, which specifies the binomial distribution of the outcome and employs an identity link function, allows the decomposition model to hold and generates reliable coefficient estimates that remain consistent regardless of the choice of reference groups [41].

The steps involved in the decomposition analysis can be outlined as follows: In the first step, a regression model is applied to the health outcome variable for all $x_{k}$ to obtain the marginal effect of determinants $\beta_{\dot{k}}^{m}$. These marginal effects indicate the relationship between the explanatory variables (contributing factors) and the outcome variables (utilization of skilled birth attendant). In the second step, we calculated the elasticity of the outcome variables for each x (${\overset{―}{x}}_{k}$). This measures how the outcome variable responds to changes in its determinant variable $\left(\frac{\beta_{\dot{k}}^{m},\;{\overset{―}{x}}_{k}}{\mu }\right)$. In the third step, the CI is computed for the health outcome variable and each explanatory variable. In the fourth step, the contribution of each explanatory variables to the overall ECI is determined by multiplying the elasticity of each factor by its concentration index $\left(\frac{\beta_{\dot{k}}^{m},\;{\overset{―}{x}}_{k}}{\mu }\right)CI_{k}$. Finally, the percentage contribution of each explanatory variable to the overall inequality is determined by dividing its contribution by the ECI and multiplying by one hundred [34,39]. Stata version 17 statistical software was used for coding and data analysis.

### Ethical approval and consent to participate

We did not require ethical clearance for our study because we used data from PMA Ethiopia. To access the data, we completed an online registration process and submitted requests to the PMA data manager. After receiving approval, we downloaded the dataset from the PMA online archive located at www.pmadata.org. All procedures were followed in compliance with the Helsinki Declaration.

## Results

### Background characteristics of study participants

The study included a total weighted sample of 1966 postpartum women. About 48% of the women were in the 25–34 age categories. Nearly 20% of women were from the poorest households, and 31.5% of women had no education. Additionally, 75.5% of the women were rural residents, while 52.3% of women were from Oromia regions. Furthermore, 36.4% of women attended four or more ANC visits, whereas only 10.1% utilized maternity waiting homes before childbirth (Table 1).

**Table 1:** Characteristics of study participants and percentage of mothers attained by SBA in Ethiopia (N = 1966; PMA Ethiopia 2022).

Characteristics of study participants	Category	Study participants		Mother attained by SBA during childbirth.	
		Weighted Frequency	Percent	Weighted Frequency	Percent
Age of mother	15-24	716	36.4	470	65.7
	25-34	944	48.0	577	61.1
	35-49	306	15.6	163	53.4
Education level of the mother	no education	620	31.5	255	41.1
	Primary and secondary education	1195	60.8	808	67.6
	Higher education	151	7.7	147	97.8
Respondent’s religion	Orthodox	653	33.2	454	69.6
	Protestant	571	29.1	329	57.6
	Muslim	706	35.9	413	58.5
	Others	36	1.8	14	37.9
Marital status of the mother	Married	1870	95.1	1148	61.4
	Single	96	4.9	62	64.7
Place of residence	Rural	1484	75.5	761	51.3
	Urban	482	24.5	449	93.2
Administrative regions of Ethiopia	Addis Ababa City	92	4.7	91	99.2
	Amhara	413	21.0	292	70.7
	Oromia	1029	52.3	578	57.6
	SNNP	432	22.0	249	56.2
Antenatal care visits	Less than four visit	1251	63.6	628	50.2
	Four or more visit	715	36.4	582	81.5
Use of maternal waiting homes	No	1767	89.9	1021	57.8
	Yes	199	10.1	189	94.9
Wealth index of the household	Quintile 1 (Poorest)	391	19.9	125	32.1
	Quintile 2	395	20.1	167	42.3
	Quintile 3	389	19.8	221	56.7
	Quintile 4	396	20.1	317	80.1
	Quintile 5 (richest)	395	20.1	380	96.1

SNNP: Southern Nations, Nationalities, and Peoples’, Other includes Catholic, traditional.

### Utilization of skilled birth attendants

This study reported that the utilization of SBA among postpartum women in Ethiopia was 61.6% (95% CI: 59.0–64.0). The coverage of SBA among women with higher education was 97.8%, which is higher than 41.1% among women with no education. The utilization of SBA varied across wealth quantiles, which means higher utilization was reported among women with higher household economic status. For instance, the utilization of SBA was 32.1% among the poorest quintiles and 96.1% among the richest quintiles.

In this study, urban-rural disparities were also reported in the use of SBA. The SBA was 51.3% and 93.2% among rural and urban residents, respectively. Substantial inequalities in SBA utilization were also reported across administrative regions (subnational region) of Ethiopia. It was higher in Addis Ababa city, 99.2%, followed by 70.7% in Amhara, 57.6% in SNNP, and 56.2% in the Oromia region.

We observed significant disparities in the utilization of SBA between maternity waiting homes users and non-user women. Among women who used maternity waiting homes, the skilled birth attendant’s utilization was 94.9%, while for those who did not use MWHs, the SBA was 57.1%. Additionally, for women who had four or more ANC visits, the utilization of SBA was 81.5%. On the other hand, our study also found that there was a slight difference in the utilization of SBA across age categories. For instance, SBA utilization was 65.7% among women aged 15–24, compared to 53.4% among those aged 35–49 (Table 1).

### Socioeconomic inequality in skilled birth attendant utilization

**Fig 2:**
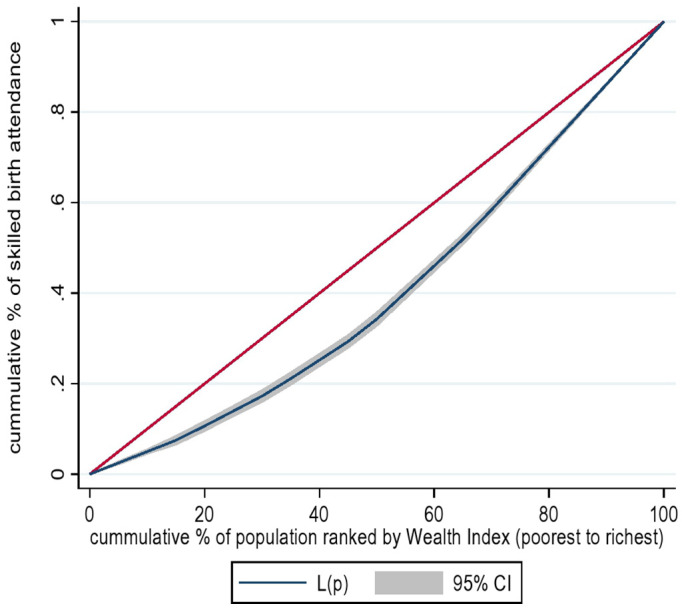
The concentration curve in utilization of SBA among postpartum women in Ethiopia, PMA Ethiopia 2022. The figure shows the concentration curve, L(p), depicted as a blue solid line, representing the cumulative proportion of skilled birth attendants across wealth-ranked population quintiles. The 45-degree red line represents the line of equality, while the shaded light gray area around the concentration curve L(p) represents the 95% confidence interval.

The concentration curve of the SBA in Ethiopia is shown in Fig 2. The concentration curve lay below the line of equality (the 45 degrees lines), illustrating that SBA was more concentrated amongst the richest group, which implies pro-rich distribution.

In this study a statistically significant inequality was reported from the inequality analysis of SBA. The overall ECI analysis of socioeconomic based health inequality in SBA in Ethiopia was 0.5308, with a standard error (SE) = 0.0398, p value < 0.001. The positive sign of concentration indices in SBA implies the existence of pro-rich socioeconomic inequalities. Women with higher socioeconomic status have a higher prevalence of utilizing SBA compared to women with lower socioeconomic status.

### Decomposing socioeconomic inequality in utilization of skulled birth attendants

The decomposition analysis reveals the contribution of different factors to the overall socioeconomic inequality in the utilization of SBA in Ethiopia. Table 2, column 3, shows the marginal effect of the contributing factors on the utilization of SBA. Contributing factors such as primary and secondary education, higher education, wealth index quintile 1, quintile 2, quantile 3, quintile 4, urban residence, living in the Oromia region, attending four or more ANC, and using maternity waiting homes had a statistically significant effect on the use SBA in Ethiopia.

**Table 2:** A decomposition analysis of socioeconomic inequality in skilled birth attendant utilization in Ethiopia, PMA Ethiopia 2022.

Contributing factors	Category	Marginal effects	Elasticity	Concentration index (ECI)	Absolute contribution	Percentage contribution	%
Age of the women	15-24	0.0193	0.0459	0.0180	0.0008	0.1560	−0.15
	25-34	−0.0177	−0.0303	0.0465	−0.0014	−0.2665	
	35-49	Base	Base	Base	Base	Base	
Education level of the women	No education	Base	Base	Base	Base	Base	16.50
	Primary and secondary education	0.1124^***^	0.2499	0.1594	0.0398	7.5111	
	Higher education	0.1723^***^	0.1672	0.2021	0.0338	6.3697	
Respondent’s religion	Orthodox	Base	Base	Base	Base	Base	−0.20
	Protestant	−0.0137	0.0031	−0.0936	−0.0002	−0.0553	
	Muslim	0.0421	0.0423	−0.0187	−0.0007	−0.1498	
	Others	−0.0514	−0.0008	−0.0234	0.0000	0.0035	
Marital status of the mother	Married	Base	Base	Base	Base	Base	−0.07
	Single	−0.0688	−0.0155	0.0269	−0.0004	−0.0789	
Place of residence	Rural	Base	Base	Base	Base	Base	16.50
	Urban	0.0610 ^*^	0.1376	0.6366	0.0876	16.5091	
Administrative regions of Ethiopia	Addis Ababa	Base	Base	Base	Base	Base	5.32
	Amhara	−0.0641	−0.1288	−0.0211	0.0027	0.5142	
	Oromia	−0.2395^**^	−0.4393	0.0219	−0.0096	−1.8144	
	SNNP	−0.1164	−0.2449	−0.1434	0.0351	6.6207	
Antenatal care visits	No	Base	Base	Base	Base	Base	15.73
	Yes	0.1153^***^	0.2256	0.3700	0.0834	15.7304	
Use of maternal waiting homes	No	Base	Base	Base	Base	Base	2.94
	Yes	0.1866 ^***^	0.1956	0.0807	0.0157	2.9745	
Wealth index of the household	Quintile 1 (Poorest)	−0.5241^***^	−0.2732	−0.6366	0.1739	32.7754	40. 34
	Quintile 2	−0.4549^***^	−0.2339	−0.3231	0.0755	14.2400	
	Quintile 3	−0.3642^***^	−0.1832	−0.0021	0.0003	0.0729	
	Quintile 4	−0.1543^***^	−0.1119	0.3196	−0.0357	−6.7435	
	Quintile 5 (richest)	Base	Base	Base	Base	Base	
Explained							94.0
Residual							6.0

Note: Significance level *** = P value < 0.001, ** = P value < 0.01, * = P value < 0.05, SNNP: Southern Nations, Nationalities, and Peoples’

Women with primary or secondary education and higher education had a 11.2% and 17.2% higher likelihood of receiving SBA, respectively, compared to women with no education. The utilization of SBA varied significantly across wealth quintiles. In the poorest quintile (quintile 1), use of SBA was 52.4% lower compared to quintile 5, the wealthiest group. This trend continued in quintile 2 with a 45.5% decrease and in quintile 3 with a 36.4% reduction. Quintile 4 had the smallest disparity, with a 15.4% decrease in SBA utilization relative to quintile 5. Urban resident women were 6% more likely to receive birth assistance from skilled healthcare providers compared to women living in rural areas. Women who attended four or more ANC visits had an 11.5% higher chance of receiving skilled birth assistance compared to their counterparts. Similarly, maternal waiting home users were 18.6% higher in receiving skilled birth assistance than non-user women. On the other hand, women residing in the Oromia region were 23.9% less likely to receive birth assistance from skilled healthcare providers compared to those in Addis Ababa.

The elasticity of each contributing factor to the inequality in SBA is presented in Table 2, column 4. Elasticity refers to the sensitivity of the SBA in response to changes in each contributing factor. Skilled birth attendant was more responsive for administrative regions, education level, wealth index, ANC four visits, use of maternity waiting homes, and place of residence. For instance, women with primary and secondary education have an elasticity value of 0.2499. This indicates that a change from no education (level one) to primary and secondary education (level two) will lead to a 24.9% increase in pro-rich socioeconomic inequality in SBA among women. The elasticity value for urban women was 0.1376, indicating that moving from a rural to an urban area would increase pro-rich socioeconomic inequality in SBA among women by 13.7%. In contrast, the Oromia administrative regions showed an elasticity of −0.4393, meaning that a change from the Oromia region to Addis Ababa city would result in a 43.9% reduction in pro-rich socioeconomic inequality among SBA.

The concentration index of the contributing factors is presented in Table 2, column 5. This index indicates both the degree and the direction of socioeconomic inequality in the use of SBA in each contributing factor. The contributing factors: age of the women 25−34 (ECI = 0.0180), age of the women 35−49 (ECI = 0.0465), primary and secondary education (ECI = 0.1594), higher education (ECI = 0.2021), single marital status (ECI = 0.0269), urban residence (ECI = 0.6366), Oromia region resident (ECI = 0.0219), having four or more ANC visits (ECI = 0.3700), use of maternal waiting homes (ECI = 0.0807), and wealth index quintile 4 (ECI = 0.3196) had a positive Erreygers concentration index, suggesting that these factors were concentrated more among women with higher socioeconomic status. In contrast, women from religious groups such as Protestants (ECI = −0.0936), Muslims (ECI = −0.0187), and other non-dominant religions (ECI = −0.0234), as well as women residing in the Amhara (ECI = −0.0211) and SNNP (ECI = −0.1434) regions, had a negative Erreygers concentration index. Additionally, those in wealth index quintiles 1 (ECI = −0.6366), quintile 2 (ECI = −0.3231), and quintile 3 (ECI = −0.0021) also demonstrated a negative Erreygers concentration index. A negative Erreygers concentration index indicates that these factors are more prevalent among socioeconomically disadvantaged women. This implies that women from lower socioeconomic status are less likely to receive birth assistance from skilled healthcare providers during childbirth.

As shown in Table 2, column 6, our study measured the contribution of each factor to the overall socioeconomic related health inequality in the utilization of SBA. If a contributing factor has a positive value, the absence of that factor would lead to a reduction in inequality in the use of SBA. Conversely, if the contributing factor has a negative value, the absence of that factor would result in an increase in the level of inequality in the use of SBA. The contributing factors included in the model explained 94.0% of the overall variability in socioeconomic inequality in the utilization of SBA in Ethiopia. The remaining 6% of the inequality observed in this study was attributed to unexplained factors or residuals.

**Fig 3:**
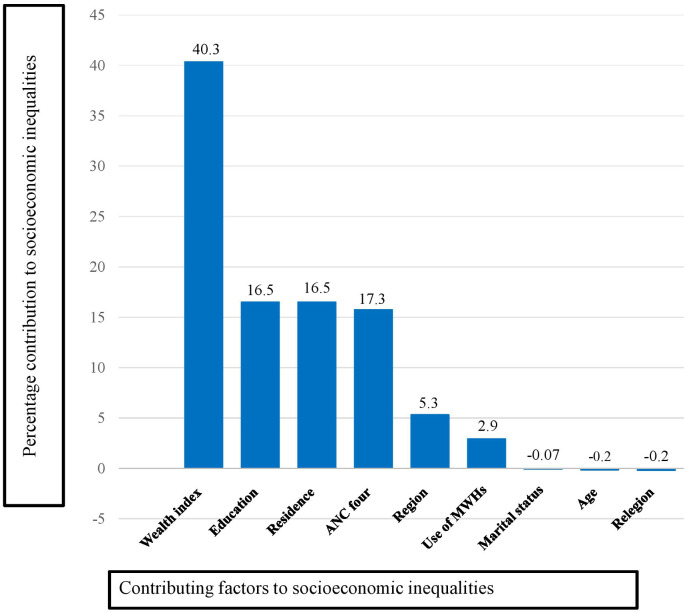
Contribution of factors to socioeconomic inequalities in skilled birth attendant utilization in Ethiopia. PMA Ethiopia 2022. The figure demonstrates the percentage contribution of different factors to the socioeconomic inequality in the utilization of skilled birth attendants, derived from a decomposition analysis of the Erreygers Concentration Index.

Fig 3 illustrated that the household wealth index (40.34%), educational level (16.50%), place of residence (16.50%), and having four or more antenatal care visits (15.73%) were the major contributors to the socioeconomic-related inequality in the utilization of SBA. On the other hand, administrative region (5.32%) and use of maternal waiting homes (2.94%) had relatively slight contributions to the inequality.

## Discussion

Measuring concentration indices and conducting decomposition analysis are essential for understanding the level of inequality and the role of various contributing factors in overall socioeconomic health related inequality. Such analysis provides valuable insight that guides policymakers and health managers in formulating effective interventions [31,42]. Therefore, this study examined the magnitude of socioeconomic inequality and the factors contributing to the utilization of SBA among postpartum women in Ethiopia. The utilization of SBA in Ethiopia was estimated at 61.6%. This finding was comparable with a study conducted in Ethiopia [7]. This result was higher than the coverage reported in the 2019 MEDHS report [18] as well as other studies carried out in Ethiopia [43,44]. However, the prevalence is below the target set by the Ethiopia Second Health Sector Transformation Plan (HSTP II) [8]. The WHO recommends that countries should attain over 90% coverage of SBA to eliminate preventable maternal deaths [3].

The overall estimated concentration index (ECI) for the utilization of SBA across different subpopulations of women was determined to be 0.5308. This finding indicated that there is statistically significant socioeconomic related health inequality in the utilization of SBA among postpartum women in Ethiopia in 2022. The positive value of the concentration index suggests a pro-rich inequality, indicating that the utilization of SBA is disproportionately higher among women with a higher socioeconomic status. The extent of pro-rich inequality observed in this study was comparatively lower than that reported in studies conducted in Nigeria [45] and Mauritania [46]. However, the pro-rich inequality in the utilization of SBA was higher when compared to studies conducted in Ethiopia [17], Zimbabwe [47], Ghana [48], Kenya [49], Tanzania [50], and Sierra Leone [51]. The discrepancy in these findings could be attributed to differences in economic development, health care policies, study periods, and countries’ strategies aimed at promoting health equality. On the other hand, this finding aligns with studies conducted in Ethiopia [7,12]. For instance, the concentration index of skilled birth attendant in 2016 was reported as 0.527 [12]. This indicates that the level of inequality in the utilization of SBA has remained unchanged between 2016 and 2022. Despite significant progress in maternal health service indicators in Ethiopia over the past 20 years, socioeconomic related health inequality in the use of maternal health services has persisted throughout this period [11,18].

The utilization of SBA has increased among women belonging to higher socioeconomic status, whereas women from disadvantaged socioeconomic status are still being neglected in accessing to skilled birth attendant services, despite having higher levels of need. Countries in Sub-Saharan Africa, such as Nigeria and Ethiopia, exhibited strong inequality in the utilization of SBA among women with different socioeconomic strata [17]. Consequently, these nations recorded a higher number of maternal deaths during the year 2020 [1]. The substantial inequality in accessing SBA likely played a role in contributing to the higher number of maternal deaths. Moreover, it hinders the advancement towards universal health coverage and meeting the maternal health service goals outlined in the Sustainable Development Goals (SDGs) [52]. The WHO has formulated five strategies to eliminate maternal mortality, with the first strategy focusing on addressing inequitable access to maternal health services, including SBA [53]. Countries with significant socioeconomic inequalities should prioritize addressing the issue of inequality in maternal health. It is essential to take action to narrow the gap between different socioeconomic groups in order to effectively reduce both maternal mortality and morbidity rates [16].

In the decomposition analysis of the concentration index, factors such as household wealth index, women’s education level, place of residence, and antenatal care were identified as the primary contributors to the overall socioeconomic-related health inequality regarding the utilization of skilled birth attendants. On the other hand, administrative regions and the use of maternal waiting homes had a minor impact on overall inequality (Fig 3).

This study found that the household wealth index was the predominant factor contributing to the overall socioeconomic related health inequality, accounting for about 40.34% of the total inequality in the utilization of SBA. Previous studies have documented that the household wealth index is an important contributing factor to the utilization of SBA [11,46,47,49,54]. Similarly, the present study demonstrated that women from wealthier households had a higher utilization of SBA services compared to women from the poorest households. Ethiopia has implemented financial protection policies, including community-based health insurance, exemption services for selected health services, and a fee waiver system, to ensure that healthcare services are accessible to all without imposing financial hardship [52]. However, despite these efforts, inequalities persist in the utilization of SBA between the poorest and richest women in the country. The user fee exemption policy in Ethiopia has played a vital role in improving the accessibility to and utilization of SBA [55]. However, it is important to note that implementing these financial protection policies alone may not be sufficient to ensure access to SBA for economically disadvantaged women [56]. The exemption of user fees for maternal health services does not cover all expenses associated with childbirth. Some costs are still borne by the women themselves. This situation can have an impact on the decision-making process of economically poor women regarding the utilization of SBA. This fact is supported by a study conducted in the Oromia region. The study highlights those women from poor households face significant financial barriers when seeking care at public hospitals due to hidden delivery costs. Specifically, non-medical expenses such as food, lodging, and transportation impose a financial burden on these women [57]. Additionally, mothers may be required to purchase medical supplies from private health facilities that are not available at public health facilities during childbirth [58]. Another important justification is that being poor significantly contributes to lower socioeconomic status, resulting in various disadvantages such as limited access to education, restricted availability of health information, living in remote areas or slum urban cities, decreased health-seeking behaviors, and reduced empowerment [59]. Collectively, these factors discourage economically disadvantaged women from accessing and utilizing SBA, placing them at a disadvantage compared to women from more affluent backgrounds [37,59].

The study revealed that approximately 16.50% of the total socioeconomic inequality in the utilization of SBA was attributed to the mothers’ educational levels (Table 2). Several studies have documented that the educational level of mothers plays a significant role in socioeconomic related inequality in the utilization of SBA [11,47,49,54]. Utilization of SBA was higher among women with higher education level compared to women with no education. The possible explanation for this finding is that as the level of education increases, women are more likely to change their attitudes towards the utilization of SBA, including their health seeking behavior towards maternal health services. With higher education, women may become more aware of the benefits of being assisted by skilled birth personnel during childbirth [60]. Additionally, women with higher levels of education generally have better access to and utilize health information effectively [61]. Furthermore, higher education is often associated with higher wealth status. Women who have attained higher education may have better job opportunities and financial freedom, as well as greater autonomy and empowerment, enabling them to afford and independently make decisions on the utilization of SBA and other maternal health services [37,62,63].

The study also showed that place of residence was the other contributing factor to socioeconomic health inequality in the utilization of SBA. It was found that the place of residence contributed 16.5% to the overall pro-rich inequality. Similar findings were reported in studies conducted in Namibia [54], Zimbabwe [47], Nigeria [45], Mauritania [46], and Ethiopia [11]. Urban women had a higher rate of utilizing SBA compared to their counterparts residing in rural areas. The urban-rural difference could be explained by the fact that urban women can easily access health facilities nearby, and transport options are readily available [58]. On the other hand, rural women face barriers such as long distances to health facilities, a lack of transportation, and geographical challenges that make it difficult to access the necessary health services [64,65]. Another possible explanation for the urban-rural difference is that women living in rural areas have limited access to education, limited empowerment, and reduced access to health information compared to their urban counterparts. These factors can contribute to the disparities in accessing SBA, as rural women may face additional challenges in seeking and utilizing maternal health services due to a lack of knowledge, awareness, and empowerment [64–67]. Moreover, disparities in infrastructure, the uneven distribution of skilled healthcare providers, and the limited availability of medical supplies may also contribute to the inequalities in the utilization of maternal health services between urban and rural residents [58,68].

In this study, having four or more ANC visits significantly contributed to the overall pro-rich socioeconomic inequality, explaining about 15.7% of the total inequality in the utilization of SBA. This result was consistent with previous studies [46,47,49]. Evidence suggests that women from socioeconomically disadvantaged groups have lower utilization rates of ANC services [69]. The literature indicates that having at least four ANC contacts has a positive effect on the utilization of SBA and institutional delivery [43,70,71]. Antenatal care benefits pregnant women by improving their knowledge about the benefits of SBA and institutional delivery services, familiarizing them with healthcare facilities, and building rapport with health care providers. It also helps reduce fear and stress and enables women to have better information about danger signs and obstetric complications. Additionally, ANC offers an opportunity for pregnant women to form informal forums where they can discuss and share information about their pregnancies and the benefits of delivering at a health facility [70]. However, access to ANC services remains low in Ethiopia, as only 43% of women have attended at least four ANC visits [18].

The administrative regions of Ethiopia accounted for approximately 5.3% of the total socioeconomic health-related inequality in the utilization of SBA. In the present study, significant subnational variation was observed in the utilization of SBA. These variations were particularly pronounced between the Addis Ababa city administration and the agrarian regions of Ethiopia. Similar findings have been documented in other studies conducted in Ethiopia [9,72]. This could be due to the fact that the regions vary in terms of health service coverage, availability of skilled healthcare providers, infrastructure, socioeconomic status, and cultural factors [9,72]. These regional differences play a role in contributing to the observed regional health inequality. Moreover, in Addis Ababa, the presence of private health facilities may have a significant impact on improving the accessibility of SBA.

In our study, the use of maternal waiting homes before the onset of labor had a minimal impact on the overall socioeconomic-related health inequality in the utilization of SBA, contributing only 2.9% to the observed inequality. However, other evidence suggests that the utilization of maternal waiting homes for pregnant women has practical implications for improving the utilization of SBA. Maternal waiting homes (MWHs) are residential facilities situated near healthcare facilities to provide accommodation for pregnant women as they await delivery [73]. They are primarily established in remote or hard-to-reach areas, addressing various barriers such as distance, geographical inaccessibility, seasonal challenges, infrastructure limitations, the means of transportation, and the cost of transportation [73,74]. It is an effective intervention in reducing geographical inequalities in accessing maternal health services, including ANC four and SBA [74,75].

Policymakers and health managers should consider a comprehensive approach that integrates both broad and specific strategies (targeted intervention). The broad approach aims to address significant social determinants of health, including economic, educational, gender, and geographic disparities [76,77]. However, it should be recognized that achieving a substantial reduction in economic, social, gender, and geographic differences within the population of developing countries may take more than ten years through this broad approach alone. Hence, it is imperative to combine a comprehensive approach with targeted interventions to achieve rapid and fundamental reductions in health-related inequalities [77]. In this study, we suggested targeted interventions, such as improving health literacy, improving the coverage of ANC four or more visits, promoting the use of maternal waiting homes, and establishing financial support programs for economically disadvantaged women. Implementing these targeted interventions can have a significant effect in the short term, contributing to the reduction of socioeconomic related health inequalities in the utilization of SBA. Furthermore, in the long term, addressing the difference in social determinants of health, particularly among women, can lead to a sustainable reduction in maternal child health inequality.

### Strengths and limitations

This study has several strengths. Firstly, we utilized data from Performance Monitoring for Action Ethiopia (PMA Ethiopia) as an alternative and new data source, rather than relying solely on the 2016 Ethiopia Demographic and Health Survey (EDHS). Secondly, the study utilized data from the most recent nationally representative survey conducted by PMA Ethiopia. Lastly, the study was conducted among women six weeks postpartum, which helps to reduce recall bias. However, the study had the following limitations: Firstly, it focused on women from three agrarian regions of Ethiopia (Amhara, Oromia, and SNNP) as well as the city administrations (Addis Ababa). However, the study did not include pastoralist regions, despite their smaller population representation. The exclusion of pastoralist regions may limit the generalizability of the findings, especially for understanding regional inequality patterns. Secondly, the PMA data did not directly capture measures of socioeconomic status such as income, spending, or consumption. As a result, we relied on an asset-based wealth index as a proxy measure for socioeconomic status.

### Conclusion

We found a strong pro-rich socioeconomic related health inequality in the utilization of SBA in Ethiopia. Household wealth index, women’s education level, place of residence, and ANC four or more visits, administrative regions, and use of maternal waiting homes were the contributing factors to the pro-rich socioeconomic related health inequality in the utilization of SBA in Ethiopia. Therefore, the government and the stakeholders need to implement targeted interventions such as improving health literacy, improving the coverage of ANC four or more visits, promoting the utilization of maternal waiting homes, and establishing financial support mechanisms for economically disadvantaged women to reduce the observed socioeconomic related health inequality in accessing and utilizing SBA.
